# Flow-Mediated Vasodilatation and Intima-Media Thickness in Patients with Coexisting Heart Failure and Diabetes Receiving Medical Therapy

**DOI:** 10.3390/diagnostics1010038

**Published:** 2011-12-08

**Authors:** Lisbeth Vestergaard Andersen, Niels Wiinberg, Christian Tuxen, Andreas Kjær

**Affiliations:** 1 Department of Clinical Physiology, Frederiksberg University Hospital, Nordre Fasanvej 57, DK-2000 Frederiksberg, Denmark; 2 Department of Cardiology, Frederiksberg University Hospital, Nordre Fasanvej 57, DK-2000 Frederiksberg, Denmark; 3 Department of Clinical Physiology, Nuclear Medicine & PET, Rigshospitalet, Copenhagen University Hospital and Cluster for Molecular Imaging, University of Copenhagen, Blegdamsvej 9, 2100 Copenhagen, Denmark

**Keywords:** diagnostic methods, ultrasound, diabetes, heart failure, atherosclerosis, endothelium, intima-media

## Abstract

**Objective:**

Intensive medical treatment of heart failure (HF) patients with diabetes may reduce the endothelial dysfunction and the accelerated atherosclerotic process seen in these patients. To study this, we investigated the endothelial function and the presence of atherosclerosis as measured by flow-mediated vasodilatation (FMD) and intima-media thickness (IMT) in intensively treated patients with coexisting HF and diabetes.

**Research Design and Method:**

FMD of the brachial artery and IMT of the common carotid arteries were determined in 26 patients with systolic HF and diabetes who were in intensive medical therapy, as well as in 19 healthy controls. The two groups were matched according to age and sex. In all subjects left ventricular ejection fraction was measured by two-dimensional echocardiography. Biochemical parameters including serum cholesterol, HDL and LDL, triglyceride, glucose, hemoglobin/hemoglobin-A1C (HbA1C), brain natriuretic peptide (BNP) and N-terminal pro-BNP were also assessed.

**Results:**

Mean FMD and IMT did not differ significantly between patients and controls. Left ventricular ejection fraction was lower in patients compared to controls (P < 0.001). The patients had a higher mean BNP, NT pro-BNP, triglyceride, HbA1C and glucose in comparison to controls. Cholesterol, HDL-cholesterol and LDL-cholesterol were lower in patients compared to controls.

**Conclusions:**

Intensively treated patients with coexisting systolic HF and diabetes seem to have normal endothelial function as measured by FMD and they have no sign of accelerated atherosclerosis as measured by IMT. This suggests a positive effect of medication on the cardiovascular alterations in this group of patients.

## 1. Introduction

The combination of type 2-diabetes and chronic heart failure (HF) is associated with high cardiovascular risk. Several retrospective analyses of large clinical trials have established that patients with coexisting diabetes and HF have an increased cardiovascular morbidity and mortality compared to non-diabetic HF-patients [1-3]. This increased cardiovascular risk is thought to result from several different mechanisms including alterations of the function and structure of the arterial wall. Disturbance of the integrity of the endothelium and the atherosclerotic process are both important factors of the pathophysiologic process in patients with HF and diabetes.

Endothelial dysfunction has been found in diabetic subjects at all durations of the disease [4]. Strong evidence has also been presented for an association between HF and impaired endothelial function determined as a decrease in flow-mediated vasodilatation (FMD) [5,6]. Furthermore, diabetes enhances the progression of atherosclerosis and thereby increases the intima-media thickness (IMT) of the arterial wall [4,7]. Enlargement of IMT of the carotid arteries is also associated with coronary artery disease [8,9]. Thus, it is now recognized that alterations of both FMD and IMT of the arterial wall are related to HF as well as to diabetes.

The consequences of these arterial alterations have been investigated in different populations and seem to contribute considerably to the cardiovascular risk. Among HF patients FMD is found to be an independent predictor of cardiac death and hospitalisation due to worsening of heart failure [10]. Similarly, it has been shown that IMT of the carotid arteries is a strong predictor of myocardial infarction and stroke in elderly adults even after statistical adjustment for traditional cardiovascular risk factors [11].

With the aim of reducing the high cardiovascular risk and prolonging the life of patients with diabetes and HF, intensive treatment with neurohormonal blockade and statins is recommended in this group of patients along with continuous glycemic control [12].

Medical therapy with ACE inhibitors has been found to increase endothelial dependent vasodilatation in diabetic subjects after four month of treatment [13]. ACE inhibitors also improve endothelial function in patients with coronary artery disease [14]. Similarly, FMD is enhanced by statin therapy in diabetic patients as well as in patients with coronary artery disease [15,16]. Glycemic control is fundamental to management of diabetes, and medical treatment with metformin and insulin regulating the glucose level have been found to improve endothelial function in diabetic subjects [17,18].

The progression of atherosclerosis in the carotid arteries is affect by medical treatment as well. Statin therapy is found to decrease carotid IMT in patients with coronary artery disease following only one year of therapy [19]. Furthermore, an association has been shown between carotid IMT and treatment of diabetic patients with ACE inhibitors [20]. Finally, glycemic control using metformin therapy attenuates increase in IMT of the common carotid arteries in patients with diabetes [21].

Therefore, intensive medical treatment of diabetic patients with HF may significantly reduce endothelial dysfunction and the atherosclerotic process.

Thus, the aim of the present investigation was to evaluate the endothelial function and presence of atherosclerosis as measured by FMD and IMT in intensively treated patients with coexisting HF and diabetes.

## 2. Material and Methods

### 2.1. Subjects

The study included 26 consecutive patients with HF and type 2-diabetes mellitus (DM) enrolled from the Outpatient Clinic of Heart Failure at Frederiksberg University Hospital. HF was defined as a previous determined left ventricular ejection fraction (LVEF) ≤ 40% measured with echocardiography and all patients were required to be in stable medical therapy for at least three months prior to the study. Patients were considered to have DM if a fasting glucose concentration above 7 mmol/L had been measured at two occasions, or if patients were in pharmacological treatment for DM. In the patient group 81% had ischemic heart disease. The remaining part of the patients had HF on the basis of atrial fibrillation or cardiomyopathy due to hypertension, excessive alcohol consumption or unrecognized cause.

The control group included 19 healthy subjects enrolled from databases of people who had participated in earlier clinical studies at Frederiksberg University Hospital. All control subjects were without recognized cardiovascular diseases and they had no history of diabetes. Control subjects using medicine affecting the cardiovascular system were excluded from the study. The two groups were matched according to age, sex and smoking.

The Study was approved by the local Ethics Committee ((KF) 01–121/04) and was carried out according to the ethical principles of the Declarations of Helsinki.

### 2.2. Ultrasound Measurements

FMD and IMT were determined using a high-resolution ultrasound-doppler-system 128 XP/10c (Acuson, Siemens Medical Solutions, Malvern, USA) with a linear 7–10 MHz transducer. All examinations were carried out in a dark, quiet and temperature controlled room in the afternoon between 12.30 am and 5.30 pm. Participants were fasting for 6 hours prior to the examination and they were asked not to smoke or use nicotine products for the same period of time. At the beginning of the examination subjects were placed in a supine resting position on an examination table for 10 min. before initiation of measurements and they remained lying down for the rest of the examination. An electrocardiogram was recorded by a monitor integrated with the ultrasound machine and the blood pressure were determined in the left brachial artery two times during the examination using oscillometric blood pressure equipment—either Dinamap 8100 (Critikon Inc., Tampa, USA) or Omron HEM-705CP (Omron, Tokyo, Japan).

At first, the left and right common carotid arteries were scanned for evaluation of IMT. A B-mode ultrasound 2D scan was obtained from the anterior/lateral side of the neck. Subjects had their head tilted a bit to the opposite side to optimise the image access to the arteries. The ultrasound probe was placed just below the bifurcation bulb and the arteries were scanned in the longitudinal plan. The depth of the scan was adjusted and the transmit focus zone set at the optimal level to show the best possible picture of the far wall of the common carotid artery. A segment of the artery was magnified using a resolution box function and the grey scale image adjusted to identify a distinct lumen-intima and media-adventitia interface of the artery wall. Ten sec. of the ultrasound image was recorded on a connected computer for later measurements of the intima-media thickness.

Subsequently subjects were placed with their right arm resting on a small table next to the examination bed in preparation for measuring endothelial function in the brachial artery. They were asked to find a comfortable position and to keep the arm as still as possible during the examinations. Measurements of FMD and nitroglycerin mediated vasodilatation (NMD) was carried out according to guidelines of the International Brachial Artery Reactivity Task Force [22]. The brachial artery was scanned in B-mode between 3 cm to 10 cm above the antecubital fossa in the longitudinal plane of the arm. In order to generate a steady picture throughout the study, the ultrasound transducer was held in a stereo tactic clamp. Great care was taken to locate the centre of the artery, which was identified when a clear image of both the anterior and posterior vessel wall intima was visible. An artery segment with a distinct lumen-intima boundary was selected and magnified. During the examination, four scans of the brachial artery were recorded for later calculation of the change in artery diameter: (1) baseline rest image, (2) during increased arterial blood flow (post-stasis), (3) a second rest image (4) following nitroglycerin administration.

Increased brachial blood flow was achieved by creating reactive hyperemia in the forearm. An inflatable cuff placed around the forearm just below the antecubital fossa was inflated by an automatic air pump. The cuff pressure was maintained at 200 mmHg for 4.5 min, which should produce maximal flow increase and maximal vessel dilatation [23]. Immediately after cuff deflation the brachial blood flow increased inducing endothelial dependent vasodilatation. Recordings of the post-stasis artery image were made from 5 sec before cuff deflation until 90 sec after.

Following more than 10 min of rest after artery occlusion the NO donor nitroglycerin was administrated sublingual to subjects (Glyceryl trinitrate 400 μg spray, Nitrolingual^®^, G. Pohl-Boskamp GmbH & CO) in order to induce endothelial independent vasodilatation. The 4th artery image was recorded from 4 min after administration of nitroglycerin. All ultrasound image recordings of the brachial artery lasted for at least 10 sec. The same operator performed all ultrasound examinations.

### 2.3. Image Processing

Two ultrasound images of the left and right common carotid arteries were selected for IMT measurements at the beginning of an ECG r-wave in two different cardiac cycles. The images with the most distinctly represented intima and media of the far wall were chosen. Using the automatic IMT-measuring tool of image evaluation software (M'Ath®, ICN-Metris, Argenteuil, France), the mean intima to media distance was calculated for the longest possible wall segment. The distance was measured from the lumen-intima interface to the media-adventitia interface determined as an echo amplitude change. The number of measuring points used for calculation of the IMT was between 5 and 333 with the mean at 118 measuring points (7.2 mm). The measuring was performed at an artery segment where atherosclerotic plaques were not visible. IMT was finally calculated as the mean IMT of the left and right common carotid arteries.

Ultrasound images from recordings of the brachial artery were also selected at the beginning of an ECG r-wave of different cardiac cycles. Two FMD-baseline pictures and two post-stasis pictures, recorded 60–70 sec. after cuff deflation, were selected as the best pictures with regard to intima clarity. Using an image-measuring program (Q-lab, Philips, Eindhoven, The Netherlands) the diameter of the brachial artery was measured in a semi-automatic way as the distance between the lumen-intima interface of the two artery walls. Measurements of the diameter were carried out in random order by an operator blinded to the identity of the images. The artery segments used for measuring were between 2.5 and 6.6 mm, depending on the quality of the image and the mean segment length was 5.0 mm (82 points).

Brachial artery diameter was determined as the mean diameter of the two measurements, and FMD was calculated as the difference between the mean diameter before and after stasis relative to the baseline diameter.

The nitroglycerin-mediated vasodilatation (NMD) was measured in the same way as FMD.

### 2.4. Biochemical Parameters

Blood samples were taken from all subjects in the morning following a fasting period with no smoking for at least 10 hours. An intravenous cannula was established in a superficial arm vein, and after 15 min of rest in supine position the blood sample was drawn. Cholesterol concentration (conc.), high density lipoprotein (HDL)-cholesterol conc. and triglyceride conc. were analysed by enzymatic methods with detection limits of 0.03, 0.04 and 0.04 mmol/L, respectively. Low density lipoprotein (LDL)-cholesterol conc. was determined from the concentration of triglyceride, cholesterol and HDL-cholesterol. The conc. of glucose were analysed using an enzymatic reference method with hexokinase and a detection limit 0.03 mmol/L. Total hemoglobin and hemoglobin-A_1C_ was determined with fotometry and immunoturbidimetry, respectively. The hemoglobin/hemoglobin-A_1C_ fraction (HbA_1C_) was calculated. The measurement of HbA_1C_ had a detection limit of 3%. An electrochemiluminescens immunoassay (Elecsys 2010, Roche Diagnostics, Basel, Switzerland) with a detection range at 5–35,000 pg/mL were used for measuring the concentration of N-terminal pro-brain natriuretic peptide (NT pro-BNP). The concentration of BNP was analysed by an automated two-site sandwich immunoassay technique, using chemiluminescent technology (Bayer, Advia Centaur, Leverkusen, Germany). The assay measures the physiologically active C-terminal peptide (77–108). The sensitivity of the BNP assay was 2 pg/mL.

### 2.5. Echocardiography

Two-dimensional echocardiography was carried out for all subjects after blood samples had been drawn using a phase array ultrasound imaging system (Acuson XP128, Siemens Medical Solutions, Malvern, USA). LVEF was assessed by a nine-segment wall motion index score (WMI). Five standard projections were imaged and recorded for later measurement of EF. An experienced cardiologist blinded to the identity of subjects performed the calculation of EF from the WMI for all examined subjects. Each image was divided according to the nine-segment model and scored by visual assessment on the 5-point scale as described by *Berning et al.* [24]. The average score was determined for each segment and the average score of all 9 segments was calculated to derive the WMI. EF was calculated from this value using Berning's formula: EF = 30 WMI.

### 2.6. Medical Treatment

Information about current medication for each patient was derived from their medical record. The etiology of heart failure was also determined from the medical records.

All subjects filled out a questionnaire. Information requested included medical history, height, weight, and smoking and exercise habits.

### 2.7. Statistical Analyses

The statistical analysis software SPSS 10.0 was used for statistical analyses. All generated data was tested for normal distribution using the Kolmogorov-Smirnov test. Differences between groups were analyzed by Student's t-test for independent-samples. A chi-square-test was used for analysis of differences between categorical data. Results are expressed as mean ± standard error of the mean (SEM). A value of *P* < 0.05 was considered statistically significant.

## 3. Results

The group of patients with coexisting HF and DM and the group of control subjects did not differ significantly in mean age, and no differences were found in the distribution of men and women between the two groups. Cardiac function measured, as LVEF was significantly lower among patients compared to controls (P < 0.001). The group of patients had, in comparison to controls, a high BMI (P < 0.01). In the control group all subjects reported to be physically active, which is a higher number of active subjects compared to the group of patients (P < 0.05). The number of smokers and non-smokers did not differ between the two groups. Mean arterial pressure (MAP), diastolic BP and HR did not differ between the groups, whereas patients had a lower mean systolic BP compared to the group of controls (Table 1).

**Figure 1:**
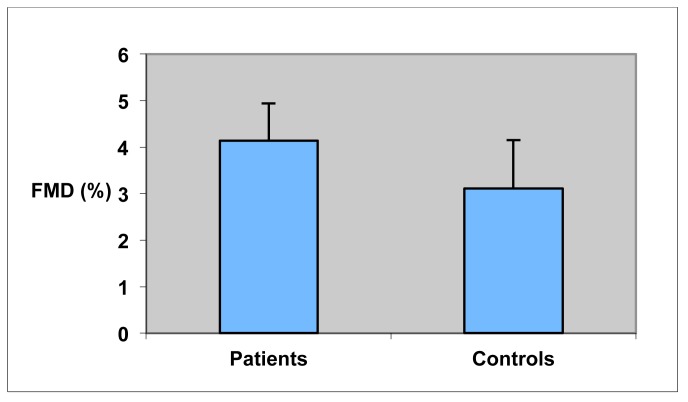

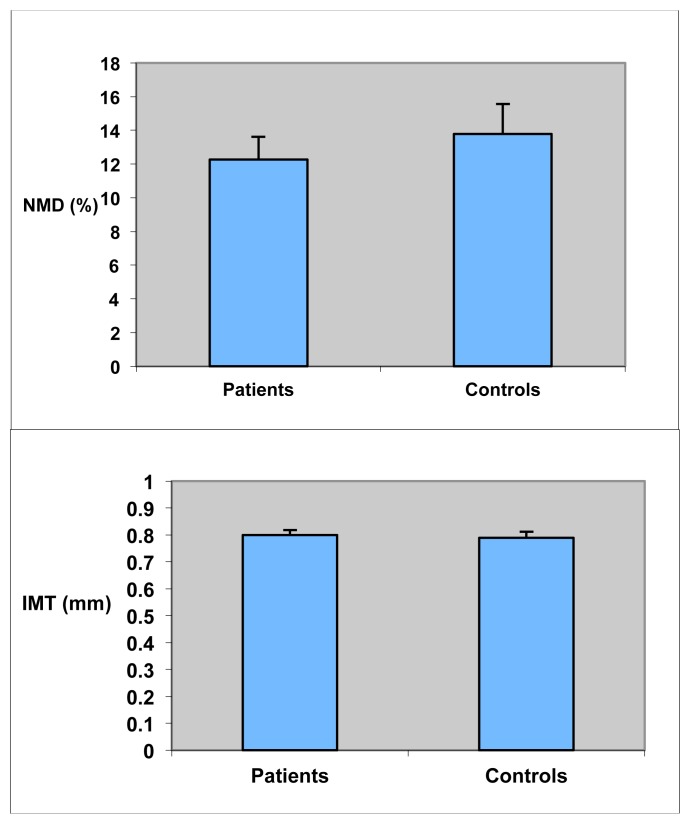
FMD and NMD of the brachial artery and mean IMT of the carotid arteries in patients and controls. Data are mean and error bars indicate SEM. The three parameters do not differ significantly between patients and controls.

FMD and IMT were successfully measured in all 45 subjects. Mean FMD of the brachial artery was found not to be significantly different between the two groups. Similarly, mean NMD of the patient group did not differ significantly from NMD among controls. The sonographic results also showed no significant difference in mean IMT of the common carotid arteries between the two groups (Figure 1). In the patient group, FMD correlated with chol/HDL-ratio (Pearson: R = −0.37; p = 0.07/spearman: R = −0.50; P = 0.01) and IMT with LDL-cholesterol (Pearson: R = 0.38; p = 0.08/spearman: R = 0.47; P = 0.02). In the control group, the only correlation was between IMT and age (Pearson: R = 0.58; p = 0.009/spearman: R = 0.62; P = 0.005).

Patients had significantly higher mean BNP, pro-BNP, triglyceride, HbA_1C_ and glucose in comparison to controls (Table 1–2). Total cholesterol, HDL-cholesterol and LDL-cholesterol were lower among patients when compared to the group of controls (Table 2). All patients were in medical therapy for HF: 88% received ACE-inhibitor or angiotensin receptor blocker, 42% beta-blocker, 77% diuretics and 12% calcium antagonist. A total of 77% received statins and 19% long-lasting nitrates. The controls did not receive any medication. In the diabetes group 6 (23%) were treated with insulin alone, 9 (35%) with oral antidiabetic drugs alone and two (8%) with both therapies. Eight patients received no antidiabetic medication. The oral antidiabetic drugs were glimepiride, glibenclamid and metromine. A total of 21 (81%) of patients received low dose aspirin, and two patients were in anti-coagulation therapy. Medication is summarized in Table 3. Eleven patients had a history of acute myocardial infarction.

## 4. Discussion and Conclusion

We found that in intensively treated patients with coexisting HF and DM, FMD and IMT did not differ from that of a group of healthy control subjects. These results indicate that endothelial function and the progression of atherosclerosis in our patients with coexisting HF and diabetes that are intensively treated, was similar to that of healthy subjects. The two groups were matched with regard to age, gender, and the number of smokers. Furthermore, there were no difference in mean arterial pressure and mean diastolic pressure between patients and controls—making the two groups comparable. It could be argued that the reason we found no difference was due a statistical type II error. However, our statistical power for detection of a relative small difference in IMT of 100 μm was 98% and for a difference of 75 μm it was 86%. Furthermore, when aiming at detecting a relative difference as seen in patients with either DM or HF [4-6], we had a statistical power of 60% for the FMD measures. Accordingly, we find that our data do indeed demonstrate that no major difference was present.

Previous studies have demonstrated a decreased FMD and enhanced IMT in patients with diabetes, and an impaired FMD in HF patients when compared to controls [4-6]. The contradictory results concerning FMD could be explained by a better condition of HF disease among patients in the present study compared to the previous, since an association has been found between impaired FMD and the severity of HF [6,25]. However, mean LVEF of the studied patients was comparable to the cardiac function of patients in the previous studies mentioned above. Among patients with diabetes, it is shown that the severity of diabetes contributes independently to carotid IMT [7] and thus, a recent diabetic debut could explain the normal IMT value for patients in the present study. However, the mean duration of diabetes in our study was more that eight years and the average HbA_1C_ was 7.9 ± 0.4% indicating an adverse diabetic status. Furthermore, ischemic heart disease had previously been established in 81% of the studied patients and it is widely recognized that patients with coronary artery disease have an increased carotid IMT compared to control subjects. Also FMD is strongly related to ischemic disease since impaired endothelial function is believed to be an initial step of the atherosclerotic process [26]. Thus, considering the severity and etiology of HF and diabetes among the studied patients, it would be expected that the group of patients had measurable alterations in FMD and IMT when compared to the control group.

All patients in the study were attached to a specialized outpatient clinic for heart failure and strictly treated according to current recommendations. Accordingly, the majority of patients were in therapy with statins, ACE inhibitors, and diuretics. Beta-blockers and oral anti-diabetic drugs were used by more than 30% of the studied patients. This intensive medical therapy may have improved the endothelial function and attenuated the atherosclerotic process of these patients with coexisting HF and diabetes—normalizing the FMD and IMT.

Inhibition of the enzyme HMG CoA reductase caused by statin medication reduces LDL-cholesterol blood concentration and may thereby influence the endothelial function, since high cholesterol blood concentration is associated with impaired FMD [27]. In the present study patients indeed had a lower LDL-Cholesterol blood concentration compared to controls—most likely due to statin medication—supporting the possibility that medical therapy with statins have contributed to the normalization of FMD in patients. Moreover, it has been shown that brachial FMD is higher in patients with coronary artery disease treated with statins compared to patients not in statin therapy and for all subjects in this treatment study FMD correlated with statin dose [15]. Statin therapy has also been found to induce an improvement of FMD in patients with type 2 diabetes [16]. Some data also support the idea that statins have a direct effect on endothelial cells independent of LDL-cholesterol level—the so-called pleiotropic effects [28], e.g., through their anti-inflammatory effects [29]. However, LDL still seems to be the single most important mechanism behind reduced cardiovascular events as found in a recent large meta analysis of 90,056 patients in 14 randomized clinical trials of statins [30]. Inhibition of HMG CoA reductase in endothelial cells has been shown to upregulate the expression and the activity of the enzyme endothelial nitrogen oxide synthase (eNOS) facilitating the syntheses of the vasodilator NO [31]. This positive effect of statins on eNOS could result in an improvement in NO mediated endothelial-dependent vasodilatation and endothelial function in general beyond the one achieved by reduction in serum LDL-cholesterol level. These data point towards a dual effect of statins on FMD. However, the association between statin therapy and FMD augmentation is not consistent regarding diabetic patients—some studies fail to show a significant improvement of FMD after statin treatment of patients with diabetes [32].

Plasma LDL-cholesterol is positively associated with carotid IMT both in healthy volunteers and among patients with coronary artery disease [33], which could indicate that LDL-lowering drugs have a decreasing effect on IMT. Indeed, a recent meta analysis of 11 trials with almost 4,000 patients did indeed find a regression or slowing of progression of IMT when statin therapy was given [34]. The effect of statins on the atherosclerotic progression has been most thoroughly investigated in relation to coronary artery disease, and statins generally seem to reduce the carotid IMT for these patients [19,35]. However, the data regarding diabetic patients is less consistent. Two years of statin therapy did not reduce carotid IMT in a study including 250 patients with diabetes and no difference was found in IMT between the treatment group and the placebo group [36].

Medical therapy with ACE inhibitors, which is known to markedly improve mortality rates in HF patients, also seems to have an effect on FMD. It has been shown that chronic administration of an ACE inhibitor increases endothelial dependent vasodilatation in HF patients as well as in diabetic subjects [13,37]. Furthermore, a slowing of carotid IMT increase has been shown in diabetic patients when treated with ACE inhibitors [20]. New evidence indicates that ACE inhibitors probably have a direct effect on the atherosclerotic process and the endothelial function. This idea is based on the fact that when lowering blood pressure to the same extent with different classes of antihypertensive drugs, only ACE inhibitors seem to improve FMD [38,39]. The antiatherogenic properties of ACE inhibitors may derive from prevention of the effects of angiotensin II, such as increase in arterial oxidative stress and vasoconstriction. Inhibition of the renin-angiotensin system also enhances formation of endothelial nitric oxide and influences inflammation and fibrinolytic balance [40,41].

The endothelial-independent vasodilatation measured as NMD did not differ between groups. This indicates that vasodilatation in the brachial artery mediated by the effect of NO on smooth muscle cells is comparable between groups.

In summary, we conclude that when patients with coexisting HF and DM are intensively treated in a specialized heart failure clinic they seem to have normal endothelial function as measured by FMD, as well as no sign of accelerated atherosclerosis as measured by IMT. Beneficial effects of, e.g., glycemic control, statins and ACE-inhibitors may contribute to the explanation.

## Figures and Tables

**Table 1:** Characteristics of patients and controls.

	**Patients (n** = **26)**	**Controls (n** = **19)**	**P-values**
Age (years)	68.8 ± 1.8	69.0 ±2.0	NS
Women (subjects)	4	7	NS
Men (subjects)	22	12	NS
EF (%)	*30.0 ± 1.8	58.2 ±0.6	0.00
BNP (pg/mL)	*72.57 ± 1.23	21.58 ± 1.21	0.000
proBNP (pg/mL)	*65.09 ± 1.30	7.56 ± 1.28	0.000
BMI (kg/m^2^)	*29.5 ± 1.0	25.9 ±0.8	0.009
MAP (mmHg)	89.9 ± 2.3	96.2 ± 2.2	NS
Systolic BP (mmHg)	*124.7 ± 3.3	136.2 ± 4.1	0.032
Diastolic BP (mmHg)	67.7 ± 2.0	71.1 ± 2.0	NS
Heart rate (beats/min)	65.2 ± 2.0	60.9 ± 2.4	NS
DM duration (years)	8.1 ± 1.6	–	
Smokers (%)	30.7	31.6	NS

Data are mean ± SEM.

* Significantly different from controls.

BNP and proBNP is calculated from statistical analyses of the ln-value.

**Table 2:** Biochemical parameters in patients and controls.

	**Patients (n** **=** **25)**	**Controls (n** **=** **19)**	***P*-values**
Glucose (mmol/L)	*8.2 ± 0.7	5.1 ± 0.1	0.001
HbA_1C_ (%)	*7.9 ± 0.4	6.0 ± 0.1	0.001
Cholesterol (mmol/L)	*4.3 ± 0.2	5.5 ± 0.2	0.001
LDL-chol. (mmol/L)	*2.2 ± 0.2	3.4 ± 0.2	0.000
HDL-chol. (mmol/L)	*1.2 ± 0.1	1.7 ± 0.1	0.000
Triglyceride (mmol/L)	*1.61 ± 1.17	0.84 ± 1.12	0.003

Data are mean ± SEM

* Significantly different from controls

Mean concentration of triglyceride is calculated from statistical analyses of the ln-value.

**Table 3:** Medical treatments of patients with HF and diabetes.

**Medicine**	**Patients in medical therapy**

Statins	77%
ACE-inhibitor	62%
Ang2-receptor antagonist	27%
Beta-receptor antagonists	42%
Ca2-antagonists	12%
Diuretics	77%
Nitrate-drugs (long lasting)	19%
Oral antidiabetica	31%
Insulin	27%
